# The genome sequence of the Orange Underwing, *Archiearis parthenias* (Linnaeus, 1761) (Lepidoptera: Geometridae)

**DOI:** 10.12688/wellcomeopenres.26261.1

**Published:** 2026-05-02

**Authors:** David C. Lees, Natural History Museum Genome Acquisition Lab, Darwin Tree of Life Barcoding Collective, Wellcome Sanger Institute Tree of Life Management, Samples and Laboratory team, Wellcome Sanger Institute Scientific Operations: Sequencing Operations, Wellcome Sanger Institute Tree of Life Core Informatics team, Tree of Life Core Informatics collective, Darwin Tree of Life Consortium

**Affiliations:** 1 Natural History Museum, London, England, UK

**Keywords:** Archiearis parthenias, Orange Underwing, genome sequence, chromosomal, Lepidoptera

## Abstract

We present a genome assembly from an individual female *Archiearis parthenias* (Orange Underwing; Arthropoda; Insecta; Lepidoptera; Geometridae). The assembly contains two haplotypes with total lengths of 528.46 megabases and 426.17 megabases. Most of haplotype 1 (99.61%) is scaffolded into 26 chromosomal pseudomolecules, including the W and Z sex chromosomes. Haplotype 2 was assembled to scaffold level. The mitochondrial genome has also been assembled, with a length of 17.24 kilobases. This assembly was generated as part of the Darwin Tree of Life project, which produces reference genomes for eukaryotic species found in Britain and Ireland.

## Species taxonomy

Eukaryota; Opisthokonta; Metazoa; Eumetazoa; Bilateria; Protostomia; Ecdysozoa; Panarthropoda; Arthropoda; Mandibulata; Pancrustacea; Hexapoda; Insecta; Dicondylia; Pterygota; Neoptera; Endopterygota; Amphiesmenoptera; Lepidoptera; Glossata; Neolepidoptera; Heteroneura; Ditrysia; Obtectomera; Geometroidea; Geometridae; Archiearinae; *Archiearis*; *Archiearis parthenias* (Linnaeus, 1761) (NCBI:txid104468).

## Background

*Archiearis parthenias* (Linnaeus, 1761), the Orange Underwing, is a striking diurnal moth in the family Geometridae. The male has a forewing length of about 16–19 mm ( Waring *et al.*, 2017) and a wingspan of about 32–35 mm ( Lepiforum, 2025). Its forewing is dark brown with a whitish to greyish central fascia internal to a subcostal eye-ring at 2/3, while the hindwing is also brown with an irregular orange submarginal area and a blackish margin, whereas it is mostly orange on the underside of both wings. *A. parthenias* is easily confused with *Boudinotiana notha* (Hübner, [1803]), which is confined to areas with Aspen ( *Populus tremula* L.). Male *B. notha* can be distinguished from *A. parthenias* by their more feathery antenna compared to the finely serrate antenna of *A. parthenias*, and an absent or less prominent central whitish forewing band ( Lepiforum, 2025; Waring *et al.*, 2017).

The Orange Underwing flies by day in March to April with a peak in mid-March ( Randle *et al.*, 2019), fluttering in the sun around the tops of birches and nectaring on sallow catkins. The male sometimes basks on open ground, resting in a triangular position. The female oviposits on twigs of birches ( *Betula* spp.), on which the greenish larva with a prominent white lateral stripe feeds nocturnally, at first on catkins and then on young leaves from April to June, spinning leaves together for a daytime shelter; the dark brown pupa with a recurved cremastral hook is formed in a silken chamber made in bark ( Lepiforum, 2025; Waring *et al.*, 2017). The moth frequents open woodland and heathland and appears to be declining in some areas of the UK ( Randle *et al.*, 2019).

The Orange Underwing is widespread across the United Kingdom – 6 411 records on the NBN Atlas ( NBN Atlas Partnership, 2025), including a historical record for Jersey in the Channel Islands – except the extreme north of Scotland ( Leverton & Cubitt, 2024), but it is absent from Ireland ( Randle *et al.*, 2019). There are 23 651 records in Europe ( GBIF Secretariat, 2025) from Spain as far east as Japan and Kamchatka in Siberia and from the southern Mediterranean to northern Scandinavia.

On BOLD (05/01/2026) the DNA barcode from the mitogenome assembly from the genome presented here (OZ321010.1) represents the Barcode Index Number (BIN) BOLD:AAC5368, which is identical to the commonest haplotype ( *n* = 21) from Europe. *A. parthenias* has an additional BIN in Europe (BOLD:ACE9095), with a pairwise distance of 1.06%. The species is most closely related to the North American species *A. infans* (Möschler, 1862) (BOLD:AAC3763), which has a *p*-distance of about 2.56% from BOLD:ACE9095. Murillo-Ramos *et al.* (2021), using a phylogeny based on COI + up to 10 protein-coding nuclear genes, found support for the following topology within the subfamily Archiearinae Fletcher, 1953: *Leucobrephos brephoides* (Walker, 1857) + ( *Boudinotiana notha* + ( *A. parthenias* + *A. infans*)). *A. infans* is also birch-feeding, while the genus *Boudinotiana* Leraut, 2002 (which includes *B. hodeberti* Leraut, 2002 from Asia), has two other European species *Boudinotiana puella* (Esper, 1787) (which feeds on *Populus* L.) and *B. touranginii* (Berce, 1870) (which feeds on *Salix purpurea* L.) ( Leraut, 2002). The genome presented here will be useful for further phylogenetic and evolutionary studies.

**Figure 1.**
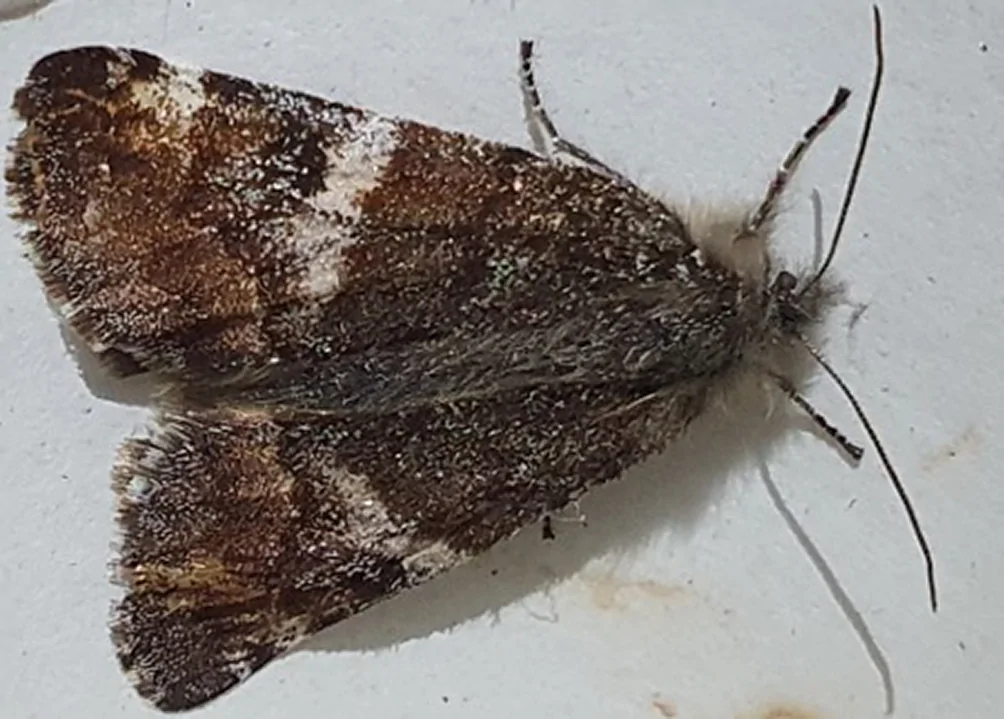
Specimen of the Orange Underwing from Stoke Common, Buckinghamshire, on 2025-04-30.

We present a chromosome-level genome sequence for *Archiearis parthenias*, the Orange Underwing. The assembly was produced using the Tree of Life pipeline using a specimen collected from Stoke Common, England, UK ( Figure 1).

## Methods

### Sample acquisition and DNA barcoding

The specimen used for genome sequencing was an adult female *Archiearis parthenias* (specimen ID NHMUK014585020, ToLID ilArcPart1), collected from Stoke Common, England, UK (latitude 51.56, longitude −0.58) on 2024-03-31. The specimen was collected and identified by David Lees (Natural History Museum).

The initial identification was verified by an additional DNA barcoding process according to the framework developed by Twyford *et al.* (2024). A small sample was dissected from the specimen and stored in ethanol, while the remaining parts were shipped on dry ice to the Wellcome Sanger Institute (WSI) (see the protocol). The tissue was lysed, the COI marker region was amplified by PCR, and amplicons were sequenced and compared to the BOLD database, confirming the species identification ( Crowley *et al.*, 2023). Following whole genome sequence generation, the relevant DNA barcode region was also used alongside the initial barcoding data for sample tracking at the WSI ( Twyford *et al.*, 2024). The standard operating procedures for Darwin Tree of Life barcoding are available on protocols.io.

### Nucleic acid extraction

Detailed protocols for nucleic acid extraction developed at the Wellcome Sanger Institute (WSI) Tree of Life Core Laboratory are available on protocols.io ( Howard *et al.*, 2025). The ilArcPart1 sample was weighed and triaged to determine the appropriate extraction protocol. Tissue from the abdomen was homogenised by powermashing using a PowerMasher II tissue disruptor. High molecular weight (HMW) DNA was extracted in the WSI Scientific Operations core using the Automated MagAttract v2 protocol. Sheared DNA was purified by automated SPRI (solid-phase reversible immobilisation). The concentration of the sheared and purified DNA was assessed using a Nanodrop spectrophotometer and Qubit Fluorometer using the Qubit dsDNA High Sensitivity Assay kit. Fragment size distribution was evaluated by running the sample on the FemtoPulse system. For this sample, the final post-shearing DNA had a Qubit concentration of 41.74 ng/μL and a yield of 2 136.17 ng, with a fragment size of 13.9 kb. The Genomic Quality Number (GQN) was 6.2.

### PacBio HiFi library preparation and sequencing

Library preparation and sequencing were performed at the WSI Scientific Operations core. Libraries were prepared using the SMRTbell Prep Kit 3.0 (Pacific Biosciences, California, USA), following the manufacturer’s instructions. The kit includes reagents for end repair/A-tailing, adapter ligation, post-ligation SMRTbell bead clean-up, and nuclease treatment. Size selection and clean-up were performed using diluted AMPure PB beads (Pacific Biosciences). DNA concentration was quantified using a Qubit Fluorometer v4.0 (ThermoFisher Scientific) and the Qubit 1X dsDNA HS assay kit. Final library fragment size was assessed with the Agilent Femto Pulse Automated Pulsed Field CE Instrument (Agilent Technologies) using the gDNA 55 kb BAC analysis kit.

The sample was sequenced on a Revio instrument (Pacific Biosciences). The prepared library was normalised to 2 nM, and 15 μL was used for making complexes. Primers were annealed and polymerases bound to generate circularised complexes, following the manufacturer’s instructions. Complexes were purified using 1.2X SMRTbell beads, then diluted to the Revio loading concentration (200–300 pM) and spiked with a Revio sequencing internal control. The sample was sequenced on a Revio 25 M SMRT cell. The SMRT Link software (Pacific Biosciences), a web-based workflow manager, was used to configure and monitor the run and to carry out primary and secondary data analysis.

### Hi-C

* **Sample preparation and crosslinking**

The Hi-C sample was prepared from 20–50 mg of frozen head tissue of the ilArcPart1 sample using the Arima-HiC v2 kit (Arima Genomics). Following the manufacturer’s instructions, tissue was fixed and DNA crosslinked using TC buffer to a final formaldehyde concentration of 2%. The tissue was homogenised using the Diagnocine Power Masher-II. Crosslinked DNA was digested with a restriction enzyme master mix, biotinylated, and ligated. Clean-up was performed with SPRISelect beads before library preparation. DNA concentration was measured with the Qubit Fluorometer (Thermo Fisher Scientific) and Qubit HS Assay Kit. The biotinylation percentage was estimated using the Arima-HiC v2 QC beads.

***Hi-C library preparation and sequencing***

Biotinylated DNA constructs were fragmented using a Covaris E220 sonicator and size selected to 400–600 bp using SPRISelect beads. DNA was enriched with Arima-HiC v2 kit Enrichment beads. End repair, A-tailing, and adapter ligation were carried out with the NEBNext Ultra II DNA Library Prep Kit (New England Biolabs), following a modified protocol where library preparation occurs while DNA remains bound to the Enrichment beads. Library amplification was performed using KAPA HiFi HotStart mix and a custom Unique Dual Index (UDI) barcode set (Integrated DNA Technologies). Depending on sample concentration and biotinylation percentage determined at the crosslinking stage, libraries were amplified with 10–16 PCR cycles. Post-PCR clean-up was performed with SPRISelect beads. Libraries were quantified using the AccuClear Ultra High Sensitivity dsDNA Standards Assay Kit (Biotium) and a FLUOstar Omega plate reader (BMG Labtech).

Prior to sequencing, libraries were normalised to 10 ng/μL. Normalised libraries were quantified again to create equimolar and/or weighted 2.8 nM pools. Pool concentrations were checked using the Agilent 4200 TapeStation (Agilent) with High Sensitivity D500 reagents before sequencing. Sequencing was performed using paired-end 150 bp reads on the Illumina NovaSeq X.

### Genome assembly

Prior to assembly of the PacBio HiFi reads, a database of *k*-mer counts ( *k* = 31) was generated from the filtered reads using FastK. GenomeScope2 ( Ranallo-Benavidez *et al.*, 2020) was used to analyse the *k*-mer frequency distributions, providing estimates of genome size, heterozygosity, and repeat content.

The HiFi reads were assembled using Hifiasm in Hi-C phasing mode ( Cheng *et al.*, 2021, 2022), producing two haplotypes. Hi-C reads ( Rao *et al.*, 2014) were mapped to the primary contigs using bwa-mem2 ( Vasimuddin *et al.*, 2019). Contigs were further scaffolded with Hi-C data in YaHS ( Zhou *et al.*, 2023), using the --break option for handling potential misassemblies. The scaffolded assemblies were evaluated using Gfastats ( Formenti *et al.*, 2022), BUSCO ( Manni *et al.*, 2021) and MerquryFK ( Rhie *et al.*, 2020).

The mitochondrial genome was assembled using MitoHiFi ( Uliano-Silva *et al.*, 2023).

### Assembly curation

The assembly was decontaminated using the Assembly Screen for Cobionts and Contaminants ( ASCC) pipeline. TreeVal was used to generate the flat files and maps for use in curation. Manual curation was conducted primarily in PretextView and HiGlass ( Kerpedjiev *et al.*, 2018). Scaffolds were visually inspected and corrected as described by Howe *et al*. (2021). Manual corrections included three breaks and 182 joins. This reduced the scaffold count by 48.5%, increased the scaffold N50 by 7.1%, and increased the total assembly length by 8.0%. The curation process is described at https://gitlab.com/wtsi-grit/rapid-curation . PretextSnapshot was used to generate a Hi-C contact map of the final assembly.

### Assembly quality assessment

The MerquryFK tool ( Rhie *et al.*, 2020) was run in a Singularity container ( Kurtzer *et al.*, 2017) to evaluate *k*-mer completeness and assembly quality for both haplotypes using the *k*-mer database ( *k* = 31) computed prior to genome assembly. The analysis outputs included assembly QV scores and completeness statistics.

The genome was analysed using the BlobToolKit pipeline, a Nextflow implementation of the earlier Snakemake version ( Challis *et al.*, 2020). The pipeline aligns PacBio reads using minimap2 ( Li, 2018) and SAMtools ( Danecek *et al.*, 2021) to generate coverage tracks. It runs BUSCO ( Manni *et al.*, 2021) using lineages identified from the NCBI Taxonomy ( Schoch *et al.*, 2020). For the three domain-level lineages, BUSCO genes are aligned to the UniProt Reference Proteomes database ( Bateman *et al.*, 2023) using DIAMOND blastp ( Buchfink *et al.*, 2021). The genome is divided into chunks based on the density of BUSCO genes from the closest taxonomic lineage, and each chunk is aligned to the UniProt Reference Proteomes database with DIAMOND blastx. Sequences without hits are chunked using seqtk and aligned to the NT database with blastn ( Altschul *et al.*, 1990). The BlobToolKit suite consolidates all outputs into a blobdir for visualisation. The BlobToolKit pipeline was developed using nf-core tooling ( Ewels *et al.*, 2020) and MultiQC ( Ewels *et al.*, 2016), with containerisation through Docker ( Merkel, 2014) and Singularity ( Kurtzer *et al.*, 2017).

## Genome sequence report

### Sequence data

**Figure 2.**
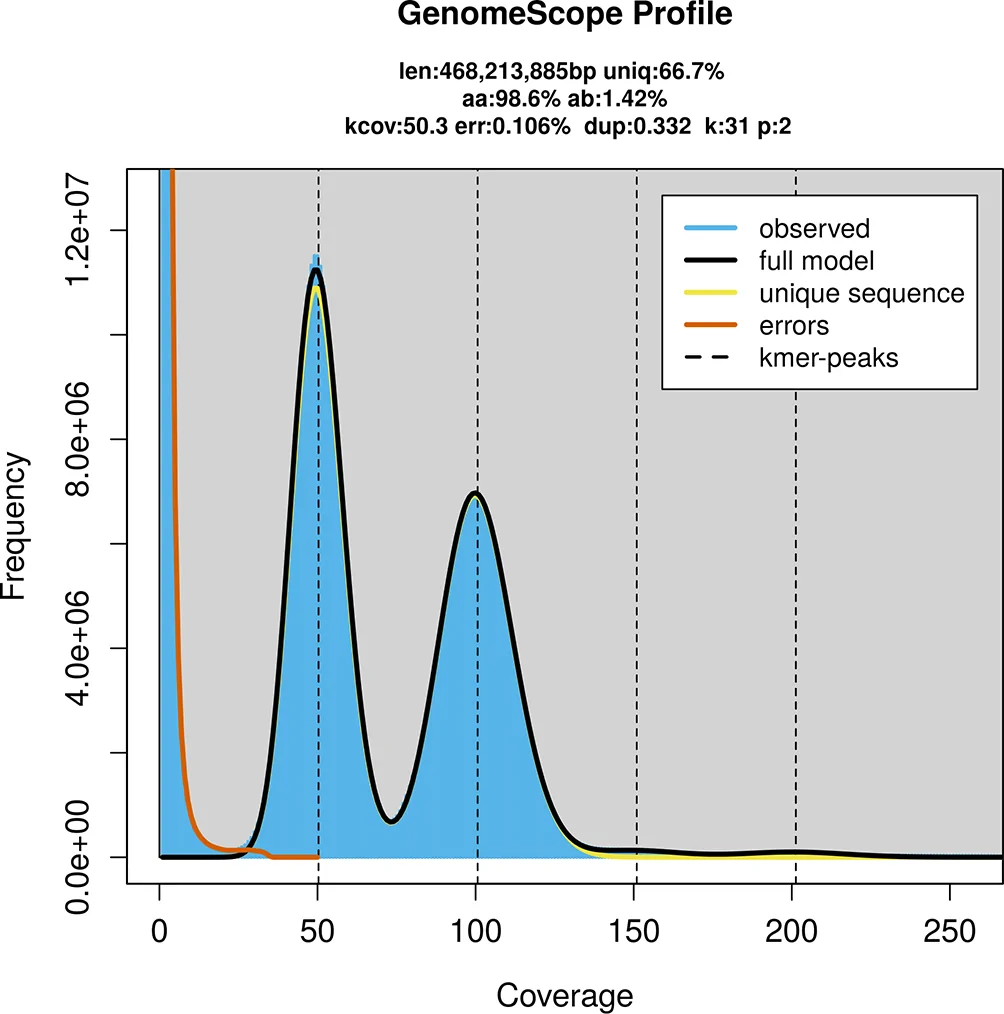
Frequency distribution of *k*-mers generated using GenomeScope2. The plot shows observed and modelled *k*-mer spectra, providing estimates of genome size, heterozygosity, and repeat content based on unassembled sequencing reads.

PacBio sequencing of the *Archiearis parthenias* specimen generated 48.87 Gb (gigabases) from 5.61 million reads, which were used to assemble the genome. GenomeScope2.0 analysis estimated the haploid genome size at 468.45 Mb, with a heterozygosity of 1.42% and repeat content of 33.28% ( Figure 2). These estimates guided expectations for the assembly. Based on the estimated genome size, the sequencing data provided approximately 101× coverage. Hi-C sequencing produced 157.37 Gb from 521.08 million reads, which were used to scaffold the assembly. Table 1 summarises the specimen and sequencing details.

**Table 1.** Specimen and sequencing data for BioProject PRJEB96584.

Platform	PacBio HiFi	Hi-C
**ToLID**	ilArcPart1	ilArcPart1
**Specimen ID**	NHMUK014585020	NHMUK014585020
**BioSample (source individual)**	SAMEA117491974	SAMEA117491974
**BioSample (tissue)**	SAMEA117492056	SAMEA117492046
**Tissue**	abdomen	head
**Instrument**	Revio	Illumina NovaSeq X
**Run accessions**	ERR15499482	ERR15500441
**Read count total**	5.61 million	521.08 million
**Base count total**	48.87 Gb	157.37 Gb

### Assembly statistics

The genome was assembled into two haplotypes using Hi-C phasing. Haplotype 1 was curated to chromosome level, while haplotype 2 was assembled to scaffold level. The final assembly has a total length of 528.46 Mb in 87 scaffolds, with 188 gaps, and a scaffold N50 of 20.61 Mb ( Table 2).

**Table 2.** Genome assembly statistics.

Genome assembly	Haplotype 1	Haplotype 2
**Assembly name**	ilArcPart1.hap1.1	ilArcPart1.hap2.1
**Assembly accession**	GCA_976915725.1	GCA_976918345.1
**Assembly level**	chromosome	scaffold
**Span (Mb)**	528.46	426.17
**Number of chromosomes**	26	scaffold-level
**Number of contigs**	275	68
**Contig N50**	18.19 Mb	18.19 Mb
**Number of scaffolds**	87	61
**Scaffold N50**	20.61 Mb	18.19 Mb
**Longest scaffold length (Mb)**	56.91	-
**Sex chromosomes**	W and Z	-
**Organelles**	Mitochondrion: 17.24 kb	-

**Figure 3.**
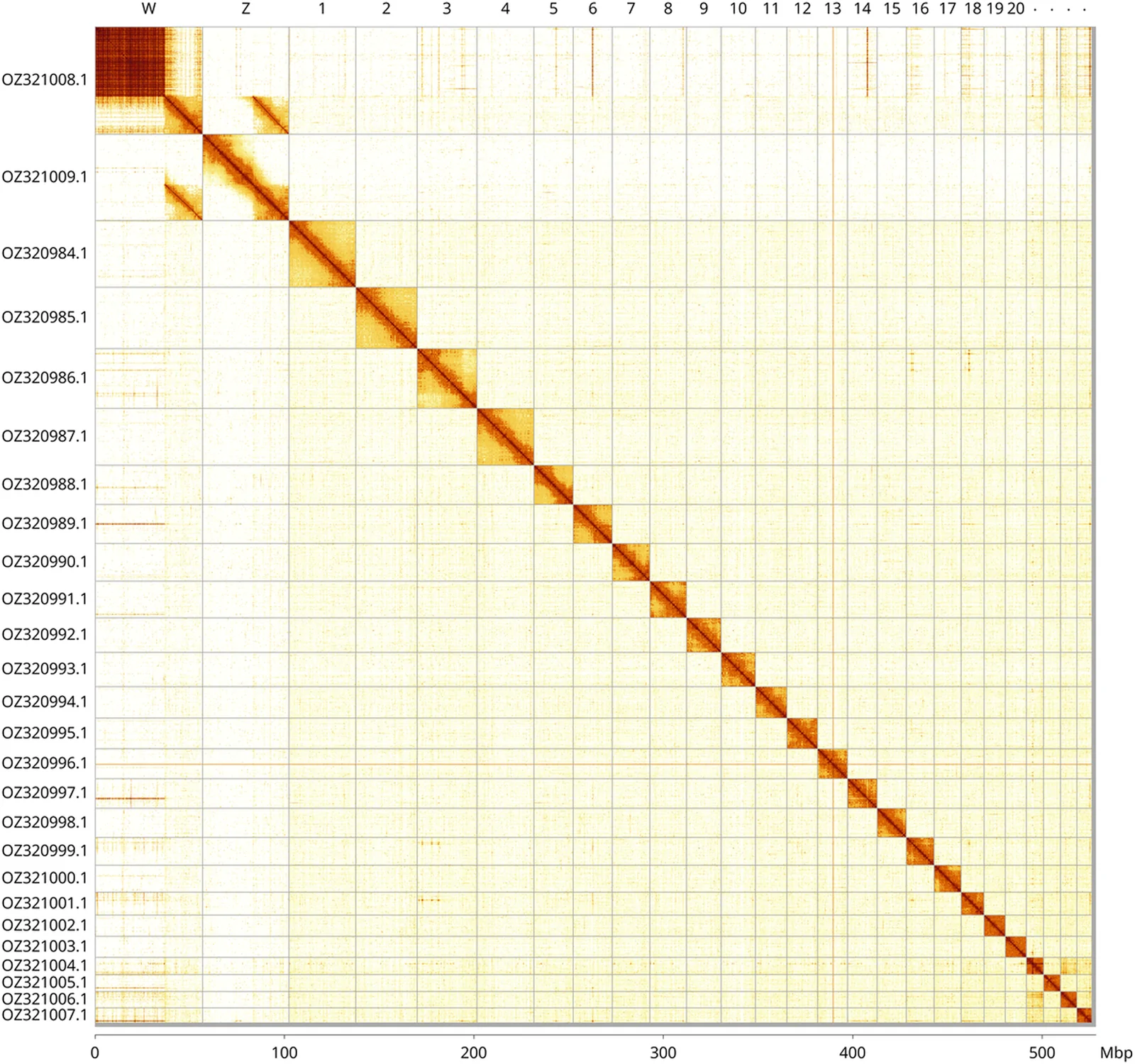
Hi-C contact map of the *Archiearis parthenias* genome assembly. Assembled chromosomes are shown in order of size and labelled along the axes, with a megabase scale shown below. The plot was generated using PretextSnapshot.

Most of the haplotype 1 assembly sequence (99.61%) was assigned to 26 chromosomal-level scaffolds, representing 24 autosomes and the W and Z sex chromosomes. These chromosome-level scaffolds, confirmed by Hi-C data, are named according to size ( Figure 3; Table 3). During curation we noted that the order and orientation of the W chromosome is uncertain from the start to 36.8 Mb.

**Table 3.** Chromosomal pseudomolecules in the haplotype 1 genome assembly of *Archiearis parthenias* ilArcPart1.

INSDC accession	Molecule	Length (Mb)	GC%
OZ320984.1	1	35.17	39
OZ320985.1	2	32.49	38.50
OZ320986.1	3	31.48	39
OZ320987.1	4	30.19	39
OZ320988.1	5	20.69	39
OZ320989.1	6	20.61	39
OZ320990.1	7	19.92	39
OZ320991.1	8	19.24	39
OZ320992.1	9	18.26	38.50
OZ320993.1	10	18.19	38.50
OZ320994.1	11	16.61	39
OZ320995.1	12	16.22	39
OZ320996.1	13	15.73	39
OZ320997.1	14	15.63	39.50
OZ320998.1	15	15.45	39.50
OZ320999.1	16	14.62	39
OZ321000.1	17	14.27	39.50
OZ321001.1	18	12.13	39.50
OZ321002.1	19	11.24	39
OZ321003.1	20	11.07	39.50
OZ321004.1	21	9.15	42
OZ321005.1	22	8.82	39.50
OZ321006.1	23	8.64	40.50
OZ321007.1	24	8.08	41
OZ321008.1	W	56.91	39.50
OZ321009.1	Z	45.59	38.50

The mitochondrial genome was also assembled (length 17.24 kb, OZ321010.1). This sequence is included as a contig in the multifasta file of the genome submission and as a standalone record.

### Assembly quality metrics

**Figure 4.**
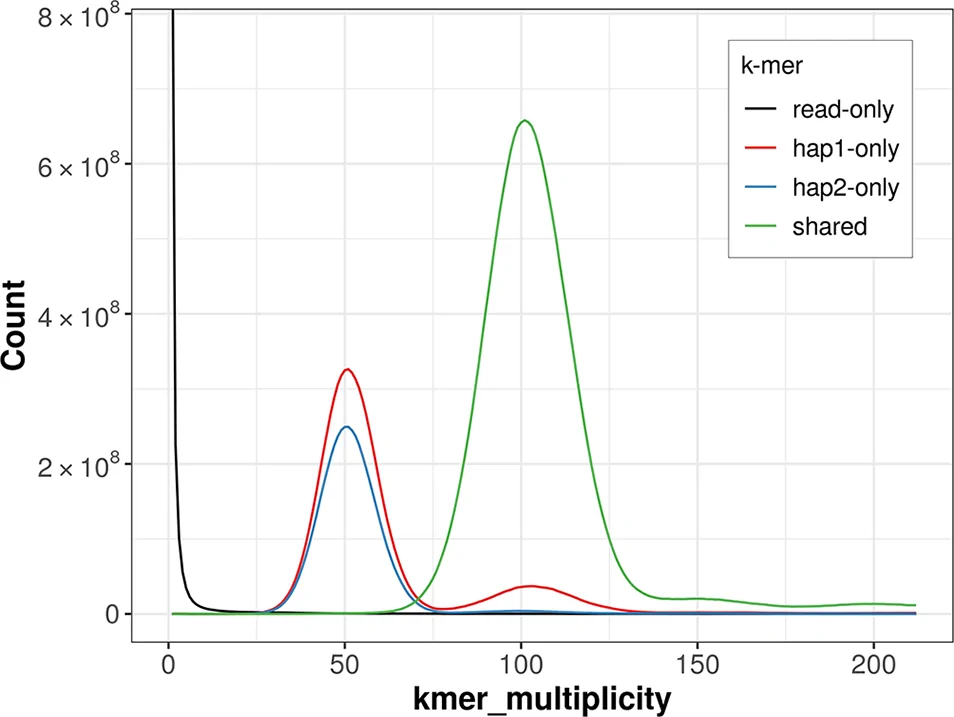
Evaluation of *k*-mer completeness using MerquryFK. This plot illustrates the recovery of *k*-mers from the original read data in the final assemblies. The horizontal axis represents *k*-mer multiplicity, and the vertical axis shows the number of *k*-mers. The black curve represents *k*-mers that appear in the reads but are not assembled. The green curve corresponds to *k*-mers shared by both haplotypes, and the red and blue curves show *k*-mers found only in one of the haplotypes.

For haplotype 1, the estimated QV is 70.4, and for haplotype 2, 74.1. When the two haplotypes are combined, the assembly achieves an estimated QV of 71.7. The *k*-mer completeness is 77.58% for haplotype 1, 68.49% for haplotype 2, and 99.63% for the combined haplotypes ( Figure 4).

**Figure 5.**
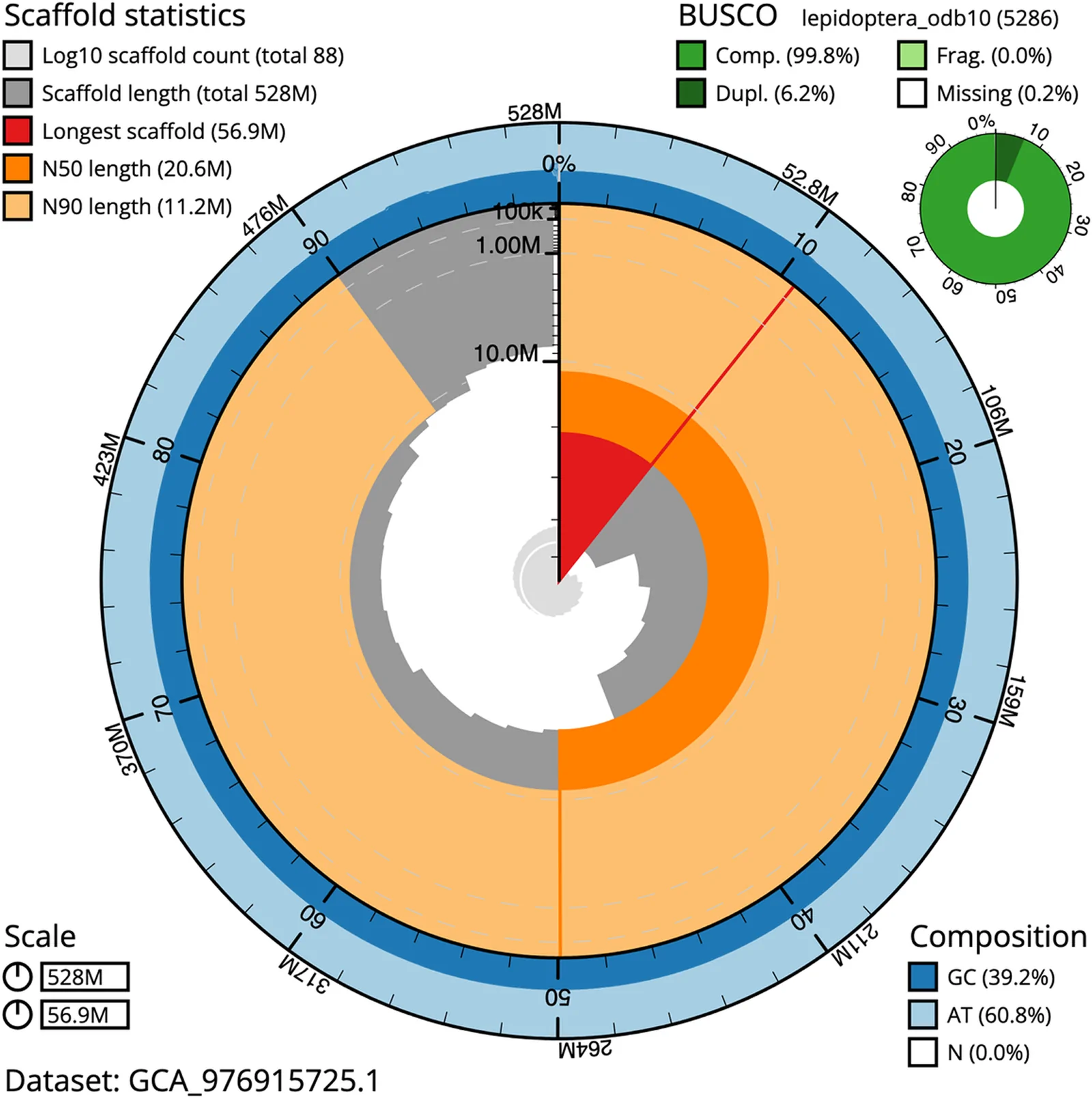
Assembly metrics for ilArcPart1.hap1.1. The BlobToolKit snail plot provides an overview of assembly metrics and BUSCO gene completeness. The circumference represents the length of the whole genome sequence, and the main plot is divided into 1 000 bins around the circumference. The outermost blue tracks display the distribution of GC, AT, and N percentages across the bins. Scaffolds are arranged clockwise from longest to shortest and are depicted in dark grey. The longest scaffold is indicated by the red arc, and the deeper orange and pale orange arcs represent the N50 and N90 lengths. A light grey spiral at the centre shows the cumulative scaffold count on a logarithmic scale. A summary of complete, fragmented, duplicated, and missing BUSCO genes in the set is presented at the top right. An interactive version of this figure can be accessed on the BlobToolKit viewer.

**Figure 6.**
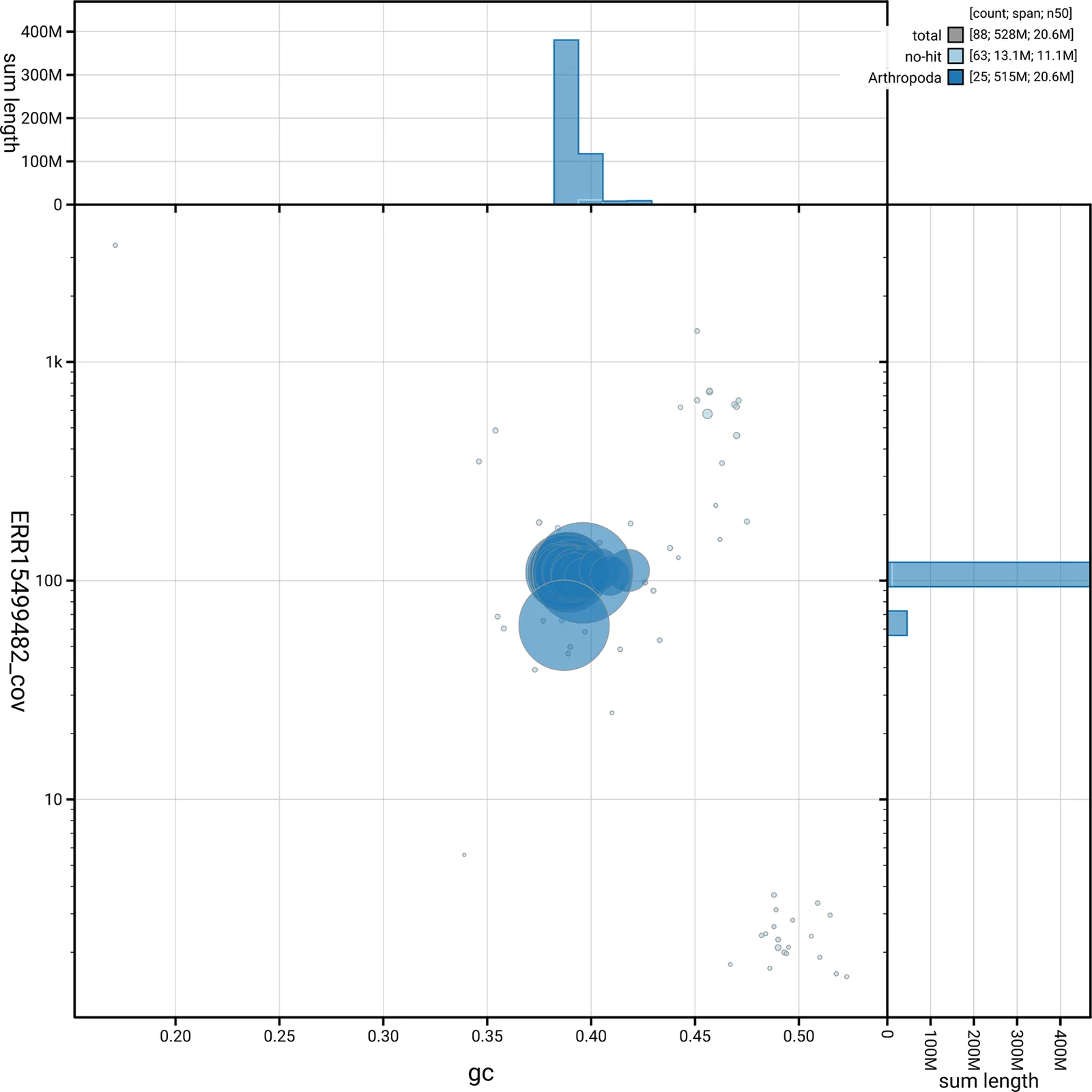
BlobToolKit blob plot for ilArcPart1.hap1.1. The plot shows base coverage (vertical axis) and GC content (horizontal axis). The circles represent scaffolds, with the size proportional to scaffold length and the colour representing phylum membership. The histograms along the axes display the total length of sequences distributed across different levels of coverage and GC content. An interactive version of this figure is available on the BlobToolKit viewer.

BUSCO analysis using the lepidoptera_odb10 reference set ( *n* = 5 286) identified 99.8% of the expected gene set (single = 93.6%, duplicated = 6.2%) in haplotype 1. For haplotype 2, BUSCO v.6.0.0 analysis identified 88.7% of the expected gene set (single = 88.2%, duplicated = 0.5%). The snail plot in Figure 5 summarises the scaffold length distribution and other assembly statistics for haplotype 1. The blob plot in Figure 6 shows the distribution of scaffolds by GC proportion and coverage for haplotype 1.

Table 4 lists the assembly metric benchmarks adapted from Rhie *et al.* (2021) and the Earth BioGenome Project Report on Assembly Standards September 2024. The EBP metric, calculated for the haplotype 1, is **7.C.Q70**, meeting the recommended reference standard.

**Table 4.** Earth Biogenome Project summary metrics for the *Archiearis parthenias* assembly.

Measure	Value	Benchmark
EBP summary (haplotype 1)	7.C.Q70	6.C.Q40
Contig N50 length	18.19 Mb	≥ 1 Mb
Scaffold N50 length	20.61 Mb	= chromosome N50
Consensus quality (QV)	Haplotype 1: 70.4; haplotype 2: 74.1; combined: 71.7	≥ 40
*k*-mer completeness	Haplotype 1: 77.58%; Haplotype 2: 68.49%; combined: 99.63%	≥ 95%
BUSCO	C:99.8% [S:93.6%, D:6.2%], F:0.0%, M:0.2%, n:5 286	S > 90%; D < 5%
Percentage of assembly assigned to chromosomes	99.61%	≥ 90%

**Notes:** The EBP summary uses log10(Contig N50); chromosome-level (C) or log10(Scaffold N50); Q (Merqury QV). BUSCO: C = complete; S = single-copy; D = duplicated; F = fragmented; M = missing; n = orthologues

**Table 5.** Software versions and sources.

Software	Version	Source
BLAST	2.14.0	ftp://ftp.ncbi.nlm.nih.gov/blast/executables/blast+/
BlobToolKit	4.4.6	https://github.com/blobtoolkit/blobtoolkit
BUSCO	6.0.0	https://gitlab.com/ezlab/busco
bwa-mem2	2.2.1	https://github.com/bwa-mem2/bwa-mem2
DIAMOND	2.1.8	https://github.com/bbuchfink/diamond
fasta_windows	0.2.4	https://github.com/tolkit/fasta_windows
FastK	1.1	https://github.com/thegenemyers/FASTK
GenomeScope2.0	2.0.1	https://github.com/tbenavi1/genomescope2.0
Gfastats	1.3.6	https://github.com/vgl-hub/gfastats
Hifiasm	0.25.0-r726	https://github.com/chhylp123/hifiasm
HiGlass	1.13.4	https://github.com/higlass/higlass
MerquryFK	1.1.2	https://github.com/thegenemyers/MERQURY.FK
Minimap2	2.28-r1209	https://github.com/lh3/minimap2
MitoHiFi	3	https://github.com/marcelauliano/MitoHiFi
MultiQC	1.14; 1.17 and 1.18	https://github.com/MultiQC/MultiQC
Nextflow	24.10.4	https://github.com/nextflow-io/nextflow
PretextSnapshot	0.0.5	https://github.com/sanger-tol/PretextSnapshot
PretextView	1.0.3	https://github.com/sanger-tol/PretextView
samtools	1.21	https://github.com/samtools/samtools
sanger-tol/ascc	0.1.0	https://github.com/sanger-tol/ascc
sanger-tol/blobtoolkit	v0.9.0	https://github.com/sanger-tol/blobtoolkit
sanger-tol/curationpretext	1.4.2	https://github.com/sanger-tol/curationpretext
Seqtk	1.3	https://github.com/lh3/seqtk
Singularity	3.9.0	https://github.com/sylabs/singularity
TreeVal	1.4.0	https://github.com/sanger-tol/treeval
YaHS	1.2.2	https://github.com/c-zhou/yahs

## Data Availability

European Nucleotide Archive: Archiearis parthenias. Accession number PRJEB96584. The genome sequence is released openly for reuse. The *Archiearis parthenias* genome sequencing initiative is part of the Darwin Tree of Life Project (PRJEB40665), the Sanger Institute Tree of Life Programme (PRJEB43745) and Project Psyche (PRJEB71705). All raw sequence data and the assembly have been deposited in INSDC databases. The genome will be annotated using available RNA-Seq data and presented through the Ensembl pipeline at the European Bioinformatics Institute. Raw data and assembly accession identifiers are reported in Tables 1 and 2. Production code used in genome assembly at the WSI Tree of Life is available at https://github.com/sanger-tol . Table 5 lists software versions used in this study.

## Author information

Contributors are listed at the following links:

• Members of the Natural History Museum Genome Acquisition Lab
• Members of the Darwin Tree of Life Barcoding collective
• Members of the Wellcome Sanger Institute Tree of Life Management, Samples and Laboratory team
• Members of Wellcome Sanger Institute Scientific Operations – Sequencing Operations
• Members of the Wellcome Sanger Institute Tree of Life Core Informatics team
• Members of the Tree of Life Core Informatics collective
• Members of the Darwin Tree of Life Consortium

## Wellcome Sanger Institute – Legal and Governance

The materials that have contributed to this genome note have been supplied by a Darwin Tree of Life Partner. The submission of materials by a Darwin Tree of Life Partner is subject to the **‘Darwin Tree of Life Project Sampling Code of Practice’**, which can be found in full on the Darwin Tree of Life website. By agreeing with and signing up to the Sampling Code of Practice, the Darwin Tree of Life Partner agrees they will meet the legal and ethical requirements and standards set out within this document in respect of all samples acquired for, and supplied to, the Darwin Tree of Life Project. Further, the Wellcome Sanger Institute employs a process whereby due diligence is carried out proportionate to the nature of the materials themselves, and the circumstances under which they have been/are to be collected and provided for use. The purpose of this is to address and mitigate any potential legal and/or ethical implications of receipt and use of the materials as part of the research project, and to ensure that in doing so we align with best practice wherever possible. The overarching areas of consideration are:

• Ethical review of provenance and sourcing of the material
• Legality of collection, transfer and use (national and international)

Each transfer of samples is further undertaken according to a Research Collaboration Agreement or Material Transfer Agreement entered into by the Darwin Tree of Life Partner, Genome Research Limited (operating as the Wellcome Sanger Institute), and in some circumstances, other Darwin Tree of Life collaborators.

## References

[ref1] Altschul SF Gish W Miller W : Basic Local Alignment Search Tool. *J. Mol. Biol.* 1990;215(3):403–410. 10.1016/S0022-2836(05)80360-2 2231712

[ref2] Bateman A Martin M-J Orchard S : UniProt: The Universal Protein Knowledgebase in 2023. *Nucleic Acids Res.* 2023;51(D1):D523–D531,D531. 10.1093/nar/gkac1052 36408920 PMC9825514

[ref3] Buchfink B Reuter K Drost H-G : Sensitive protein alignments at tree-of-life scale using DIAMOND. *Nat. Methods.* 2021;18(4):366–368,368. 10.1038/s41592-021-01101-x 33828273 PMC8026399

[ref4] Challis R Richards E Rajan J : BlobToolKit – interactive quality assessment of genome assemblies. *G3: Genes, Genomes, Genetics.* 2020;10(4):1361–1374,1374. 10.1534/g3.119.400908 32071071 PMC7144090

[ref5] Cheng H Concepcion GT Feng X : Haplotype-resolved *de novo* assembly using phased assembly graphs with Hifiasm. *Nat. Methods.* 2021;18(2):170–175,175. 10.1038/s41592-020-01056-5 33526886 PMC7961889

[ref6] Cheng H Jarvis ED Fedrigo O : Haplotype-resolved assembly of diploid genomes without parental data. *Nat. Biotechnol.* 2022;40(9):1332–1335,1335. 10.1038/s41587-022-01261-x 35332338 PMC9464699

[ref7] Crowley L Allen H Barnes I : A sampling strategy for genome sequencing the British terrestrial Arthropod fauna. *Wellcome Open Research.* 2023;8:123. 10.12688/wellcomeopenres.18925.1 37408610 PMC10318377

[ref8] Danecek P Bonfield JK Liddle J : Twelve years of SAMtools and BCFtools. *GigaScience.* 2021;10(2). 10.1093/gigascience/giab008 33590861 PMC7931819

[ref9] Ewels P Magnusson M Lundin S : MultiQC: Summarize analysis results for multiple tools and samples in a single report. *Bioinformatics.* 2016;32(19):3047–3048,3048. 10.1093/bioinformatics/btw354 27312411 PMC5039924

[ref10] Ewels PA Peltzer A Fillinger S : The nf-core framework for community-curated bioinformatics pipelines. *Nat. Biotechnol.* 2020;38(3):276–278. 10.1038/s41587-020-0439-x 32055031

[ref11] Formenti G Abueg L Brajuka A : Gfastats: Conversion, evaluation and manipulation of genome sequences using assembly graphs. *Bioinformatics.* 2022;38(17):4214–4216. 10.1093/bioinformatics/btac460 35799367 PMC9438950

[ref12] GBIF Secretariat: *Archiearis parthenias* (Linnaeus, 1761). 2025. Reference Source

[ref13] Howard C Denton A Jackson B : On the path to reference genomes for all biodiversity: Lessons learned and laboratory protocols created in the Sanger Tree of Life core laboratory over the first 2000 species. *bioRxiv.* 2025. 10.1101/2025.04.11.648334 PMC12548527 41129326

[ref14] Howe K Chow W Collins J : Significantly improving the quality of genome assemblies through curation. *GigaScience.* 2021;10(1). 10.1093/gigascience/giaa153 33420778 PMC7794651

[ref15] Kerpedjiev P Abdennur N Lekschas F : HiGlass: Web-based visual exploration and analysis of genome interaction maps. *Genome Biol.* 2018;19(1):125. 6109259. 10.1186/s13059-018-1486-1 30143029 PMC6109259

[ref16] Kurtzer GM Sochat V Bauer MW : Singularity: Scientific containers for mobility of compute. *PLOS ONE.* 2017;12(5):e0177459. 10.1371/journal.pone.0177459 28494014 PMC5426675

[ref17] Lepiforum: *Archiearis parthenias* (Linnaeus, 1761) Birch virgin. 2025. Reference Source

[ref18] Leraut P : Contribution to the study of the *Archiearis* Hübner and allied genera (Lepidoptera, Geometridae). *Annales de la Société Entomologique de France, Nouvelle Série.* 2002;107:349–358.

[ref19] Leverton R Cubitt M : *The Larger Moths of Scotland.* Triphosa Publications;2024.

[ref20] Li H : Minimap2: Pairwise alignment for nucleotide sequences. *Bioinformatics.* 2018;34(18):3094–3100. 10.1093/bioinformatics/bty191 29750242 PMC6137996

[ref21] Manni M Berkeley MR Seppey M : BUSCO update: Novel and streamlined workflows along with broader and deeper phylogenetic coverage for scoring of eukaryotic, prokaryotic, and viral genomes. *Mol. Biol. Evol.* 2021;38(10):4647–4654,4654. 10.1093/molbev/msab199 34320186 PMC8476166

[ref22] Merkel D : Docker: Lightweight Linux containers for consistent development and deployment. *Linux J.* 2014;2014(239). 10.5555/2600239.2600241

[ref23] Murillo-Ramos L Chazot N Sihvonen P : Molecular phylogeny, classification, biogeography and diversification patterns of a diverse group of moths (Geometridae: Boarmiini). *Mol. Phylogenet. Evol.* 2021;162:107198. 10.1016/j.ympev.2021.107198 Reference Source 33989807

[ref24] NBN Atlas Partnership: *Archiearis parthenias* (Linnaeus, 1761) Orange Underwing. 2025. Reference Source

[ref25] Ranallo-Benavidez TR Jaron KS Schatz MC : GenomeScope 2.0 and Smudgeplot for reference-free profiling of polyploid genomes. *Nat. Commun.* 2020;11(1):1432. 10.1038/s41467-020-14998-3 32188846 PMC7080791

[ref26] Randle Z Evans-Hill LJ Parsons MS : *Atlas of Britain & Ireland’s Larger Moths.* Newbury: Pisces Publications;2019.

[ref27] Rao SSP Huntley MH Durand NC : A 3D map of the human genome at kilobase resolution reveals principles of chromatin looping. *Cell.* 2014;159(7):1665–1680. 10.1016/j.cell.2014.11.021 25497547 PMC5635824

[ref28] Rhie A McCarthy SA Fedrigo O : Towards complete and error-free genome assemblies of all vertebrate species. *Nature.* 2021;592(7856):737–746,746. 10.1038/s41586-021-03451-0 33911273 PMC8081667

[ref29] Rhie A Walenz BP Koren S : Merqury: Reference-free quality, completeness, and phasing assessment for genome assemblies. *Genome Biol.* 2020;21(1):245. 10.1186/s13059-020-02134-9 32928274 PMC7488777

[ref30] Schoch CL Ciufo S Domrachev M : NCBI taxonomy: A comprehensive update on curation, resources and tools. *Database.* 2020;2020:baaa062. 10.1093/database/baaa062 32761142 PMC7408187

[ref31] Twyford AD Beasley J Barnes I : A DNA barcoding framework for taxonomic verification in the Darwin Tree of Life Project. *Wellcome Open Research.* 2024;9:339. 10.12688/wellcomeopenres.21143.1 39386966 PMC11462125

[ref32] Uliano-Silva M Ferreira JGRN Krasheninnikova K : MitoHiFi: A Python pipeline for mitochondrial genome assembly from PacBio high fidelity reads. *BMC Bioinformatics.* 2023;24(1):288. 10.1186/s12859-023-05385-y 37464285 PMC10354987

[ref33] Vasimuddin M Misra S Li H : Efficient architecture-aware acceleration of BWA-MEM for multicore systems. *2019 IEEE International Parallel and Distributed Processing Symposium (IPDPS).* IEEE;2019;314–24. 10.1109/IPDPS.2019.00041

[ref34] Waring P Townsend M Lewington R : *Field Guide to the Moths of Great Britain and Ireland.* Bloomsbury Publishing; Third ed 2017.

[ref35] Zhou C McCarthy SA Durbin R : YaHS: Yet another Hi-C scaffolding tool. *Bioinformatics.* 2023;39(1). 10.1093/bioinformatics/btac808 36525368 PMC9848053

