# Supplementary material for: The genome sequence of the Orange Underwing, Archiearis parthenias (Linnaeus, 1761) (Lepidoptera: Geometridae)
Source: Wellcome Open Res. 2026 May 2;11:255. [Version 1] doi: 10.12688/wellcomeopenres.26261.1 (PMC13320127; doi:10.12688/wellcomeopenres.26261.1)
Supplement: Peer review history

# Reviewer report: Reviewer response for version 1

**DOI:** 10.21956/wellcomeopenres.28923.r159057

**Published:** 2026-07-15

**Authors:** Nikoletta Andrea Nagy

**Affiliations:** 1 University of Debrecen, Debrecen, Hungary

The manuscript is well written, and the genome presented can contribute to future research on lepidopterans. I have only a few suggestions for the authors.

 - The distribution is phrased a bit strange as it first refers to Europe then sets the end to Japan and Siberia. Consider rephrasing.

 - Please include the full name of BOLD at first appearance.

 - Dilution level of AMPure PB beads is not included.

 - Size of the longest scaffold of haplotype 2 is missing from Table 2.

 - Please specify full name of QV at first appearance.

Are sufficient details of methods and materials provided to allow replication by others?

Yes

Is the rationale for creating the dataset(s) clearly described?

Yes

Are the datasets clearly presented in a useable and accessible format?

Yes

Are the protocols appropriate and is the work technically sound?

Yes

Reviewer Expertise:

arthropod genomics and transcriptomics

I confirm that I have read this submission and believe that I have an appropriate level of expertise to confirm that it is of an acceptable scientific standard.

# Reviewer report: Reviewer response for version 1

**DOI:** 10.21956/wellcomeopenres.28923.r156084

**Published:** 2026-07-15

**Authors:** Annabel Whibley

**Affiliations:** 1 The University of Auckland, Auckland, New Zealand

In this Data Note, Lees and colleagues present the reference genome assembly of *Archiearis parthenias*, known as the Orange Underwing. The sequenced individual was female, and the Z and W sex chromosomes are bundled into haplotype 1, which is scaffolded into pseudochromosomes.  An autosomal assembly of haplotype 2 has also been generated. Sample identification was supported by DNA barcoding analysis. Sequencing and assembly followed standard DToL protocols, combining PacBio HiFi and Illumina HiC datasets. Overall, the assembly quality is very high, with good supporting evaluation data.  Reporting and metadata is comprehensive, following standardized templates. The quality of haplotype 2 is noticeably a little poorer, as indicated by the lower BUSCO completeness and the retained “hap 1 only” kmer signal under the diploid peak in the Merqury analysis. I am intrigued by the contact map signal between Z and W- does this represent ambiguous mappings in the dataset or genuine interactions?

Are sufficient details of methods and materials provided to allow replication by others?

Yes

Is the rationale for creating the dataset(s) clearly described?

Yes

Are the datasets clearly presented in a useable and accessible format?

Yes

Are the protocols appropriate and is the work technically sound?

Yes

Reviewer Expertise:

Bioinformatics, Genomics

I confirm that I have read this submission and believe that I have an appropriate level of expertise to confirm that it is of an acceptable scientific standard.

# Reviewer report: Reviewer response for version 1

**DOI:** 10.21956/wellcomeopenres.28923.r159062

**Published:** 2026-06-30

**Authors:** Danilo T Amaral

**Affiliations:** 1 Universidade Federal do ABC (UFABC), Santo André, Brazil

The manuscript presents a high-quality chromosome-level genome assembly of *Archiearis parthenias. *The assembly is technically robust, with excellent quality metrics, comprehensive quality assessment, and public availability of both the raw sequencing data and the assembled genome.

 The rationale for generating this dataset is clear, the methods are appropriate and sufficiently detailed for replication, and the data are presented in a clear and accessible manner.

 I have no major concerns regarding the technical aspects of the study. As a minor suggestion, the authors could briefly discuss the differences in quality between the two haplotypes, particularly the lower completeness of haplotype 2, as this would help future users interpret the dataset. Additionally, a short comment on the uncertainty regarding the W chromosome assembly would further improve the manuscript.

Are sufficient details of methods and materials provided to allow replication by others?

Yes

Is the rationale for creating the dataset(s) clearly described?

Yes

Are the datasets clearly presented in a useable and accessible format?

Yes

Are the protocols appropriate and is the work technically sound?

Yes

Reviewer Expertise:

Bioinformatics

I confirm that I have read this submission and believe that I have an appropriate level of expertise to confirm that it is of an acceptable scientific standard.

# Reviewer report: Reviewer response for version 1

**DOI:** 10.21956/wellcomeopenres.28923.r155487

**Published:** 2026-06-03

**Authors:** Li-Wei Wu

**Affiliations:** 1 Tunghai University, Taichung, Taichung City, Taiwan

This manuscript presents a genome assembly and associated analyses for the Orange Underwing, *Archiearis parthenias*. The authors generated two haplotypes: haplotype 1 was assembled into 26 pseudochromosomes, including the W and Z chromosomes, whereas haplotype 2 showed a lower BUSCO completeness score and was therefore treated as a scaffold-level assembly. Overall, I have no major concerns regarding the data generation or analytical workflow. The genomic resources have also been deposited in public databases, which will provide a useful reference for future studies. However, given that both haplotype 1 and haplotype 2 are assembly products generated from the same sequencing dataset and processed through the same general assembly workflow, I suggest that the authors provide a more detailed comparison between the two haplotypes. For example, the manuscript could include analyses or discussion of the structural and sequence-level differences between the two assemblies, or an explanation of why the two haplotypes differ substantially in assembly quality and contiguity. Such information would help readers and future users better understand the possible biological or technical causes underlying the differences between the haplotypes, the limitations of using each assembly, and potential strategies for improving haplotype-resolved genome assembly in future work.

Are sufficient details of methods and materials provided to allow replication by others?

Yes

Is the rationale for creating the dataset(s) clearly described?

Yes

Are the datasets clearly presented in a useable and accessible format?

Yes

Are the protocols appropriate and is the work technically sound?

Yes

Reviewer Expertise:

Biogeography

I confirm that I have read this submission and believe that I have an appropriate level of expertise to confirm that it is of an acceptable scientific standard.
